# IgG1-Dominant Antibody Response Induced by Recombinant Trimeric SARS-CoV-2 Spike Protein with PIKA Adjuvant

**DOI:** 10.3390/vaccines11040827

**Published:** 2023-04-11

**Authors:** Jingxia Wang, Xinjia Mai, Yu He, Chenxi Zhu, Dapeng Zhou

**Affiliations:** Department of Immunology and Pathogen Biology, Tongji University School of Medicine, 500 Zhennan Road, Shanghai 200331, China

**Keywords:** COVID-19 vaccine, SARS-CoV-2, IgG subclass, alpha1,6 fucosyltransferase-8

## Abstract

Recombinant trimeric SARS-CoV-2 Spike protein with PIKA (polyI:C) adjuvant induces potent and durable neutralizing antibodies that protect against multiple SARS-CoV-2 variants. The immunoglobulin subclasses of viral-specific antibodies remain unknown, as do their glycosylation status on Fc regions. In this study, we analyzed immunoglobulins adsorbed by plate-bound recombinant trimeric SARS-CoV-2 Spike protein from serum of Cynomolgus monkey immunized by recombinant trimeric SARS-CoV-2 Spike protein with PIKA (polyI:C) adjuvant. The results showed that IgG1 was the dominant IgG subclass as revealed by ion mobility mass spectrometry. The average percentage of Spike protein-specific IgG1 increased to 88.3% as compared to pre-immunization. Core fucosylation for Fc glycopeptide of Spike protein-specific IgG1 was found to be higher than 98%. These results indicate that a unique Th1-biased, IgG1-dominant antibody response was responsible for the effectiveness of PIKA (polyI:C) adjuvant. Vaccine-induced core-fucosylation of IgG1 Fc region may reduce incidence of severe COVID-19 disease associated with overstimulation of FCGR3A by afucosylated IgG1.

## 1. Introduction

Several recombinant trimer-Spike protein-based vaccines have shown success in eliciting effective and durable immune responses that are protective against severe diseases and death [1,2,3,4,5,6,7,8,9,10]. NVX-CoV2373, authorized by WHO for emergency use and containing the full-length spike glycoprotein of the prototype strain plus Matrix-M adjuvant, conferred 89.7% protection against SARS-CoV-2 infection and showed high efficacy against the B.1.1.7 variant, with low incidence of severe adverse events [1,2]. SCB-2019 recombinant Spike trimer vaccine, adjuvanted with CpG-1018 and alum, provides notable protection against the entire severity spectrum of COVID-19 caused by circulating SAR-CoV-2 viruses [3]. In a prospective household contact study, vaccination with SCB-2019 reduced SARS-CoV-2 transmission compared with placebo in households and in household members [4]. SCTV01C, a trimeric spike extracellular domain (S-ECD) of SARS-CoV-2 variants Alpha (B.1.1.7) and Beta (B.1.351) with a squalene-based oil-in-water adjuvant [5,6], was authorized for emergency use in China’s 2022 pandemic of Omicron variant. The PIKA COVID-19 vaccine, a trimeric spike protein with polyI:C as adjuvant, induced high titer of neutralization antibodies toward the Omicron variant in clinical trials conducted by the UAE and Philippines [7,8,9]. These studies clearly demonstrated the feasibility of vaccinating the global population on a yearly basis to reduce viral transmission and COVID-19-associated deaths, with unlimited manufacturing capacity of recombinant proteins and adjuvants.

IgG subclasses induced by different COVID-19 vaccines have not been thoroughly characterized. Th1-biased antibody responses are considered to be more protective against viral infections. A subunit vaccine MVC-COV1901 based on the stable pre-fusion spike protein (S-2P) adjuvanted with CpG 1018 adjuvant and aluminum hydroxide induced IgG1 and IgG3 dominant IgG subclasses in humans [10]. For NVX-CoV2373 that contains the full-length spike glycoprotein of the prototype strain plus Matrix-M adjuvant, a strong bias toward this Th1 phenotype was noted, while Th2 responses (as measured by IL-5 and IL-13 cytokines) were minimal [11,12]. Painter et al. reported Th1 and Tfh cell responses induced by SARS-CoV-2 mRNA vaccination [13], measured as antigen-specific CXCR5^+^ Tfh in circulation and CXCR5^−^ Th1 (CXCR3^+^CCR6^−^). Zhang et al. reported that mRNA vaccines induced IgG1, IgG2, IgG3, and IgG4 subclasses of viral-specific antibodies, and the booster vaccination by mRNA vaccine following inactivated vaccine could increase RBD-reactive IgG1 responses to the level of three doses of mRNA vaccines [14]. van Doremalen reported that vaccination with ChAdOx1 nCoV-19 (using either a prime-only or a prime-boost regimen) induced a balanced humoral and cellular immune response of type-1 and type-2 T helper cells in rhesus macaques [15]. In human subjects, ChAdOx1 nCoV-19 induced a Th1-biased response characterized by interferon-γ and tumor necrosis factor-α cytokine secretion by CD4^+^ T cells, and antibody production predominantly of IgG1 and IgG3 subclasses [16]. 

Glycosylation of Fc portion of IgG antibodies critically regulates the stimulatory activity to Fc receptors, especially the core-fucosylation mediated by alpha1,6-fucosyltransferase 8 (FUT8) [17]. FUT8-synthesized core-fucose glycan is known to decrease the FCGR3A stimulation more than 40-fold. Independent studies by Larsen et al. and Chakraorty et al. showed that afucosylated IgG1 was associated with cytokine storm and severe cases of COVID-19 [18,19]. Afucosylation of IgG was hypothesized to be produced by B cells stimulated by enveloped viruses present on the host cell’s membrane [15]. Whether afucosylated IgG1 may be induced by vaccines is not completely characterized. Van Coillie et al. reported that mRNA vaccine transiently induced afucosylated IgGs in naïve individuals after the first and second dose of vaccines [20].

We and others previously reported the potent effect of PIKA adjuvant in inducing neutralization antibodies when combined with recombinant trimeric Spike protein of SARS-CoV-2 [7,8]. PIKA adjuvant is synthetic mismatched double-stranded RNA; one strand is a polymer of inosinic acid, while the other is a polymer of cytidylic acid (polyI:C). PolyI:C acts as toll-like receptor (TLR3) ligand to trigger anti-viral responses through recruiting IRF3 to ISRE-1 element of the IL-12p35 promoter region [21,22]. The intracellular sensors RIG-I and mda5 are the other class of receptors for polyI:C signaling [23]. In clinical studies of inactivated rabies virus vaccine adjuvanted by polyI:C, it induced more potent or non-inferior immunogenicity than commercially available vaccines in healthy adults [24,25]. Most importantly, an accelerated vaccine schedule was achieved with polyI:C adjuvanted vaccine within 7 days post vaccination [25]. 

The Cynomolgus immunoglobulin subclasses are highly conserved and function similar to human immunoglobulin subclasses; thus, they serve as an ideal model to study the vaccine efficacy and mechanisms of action [26]. In this study, we analyzed IgG subclasses in Cynomolgus monkeys vaccinated with the PIKA COVID-19 vaccine, as well as fucosylation of IgG1. 

## 2. Results and Discussion

### 2.1. PIKA Adjuvant Induced Viral-Specific IgG1 Response

**Figure 1:**
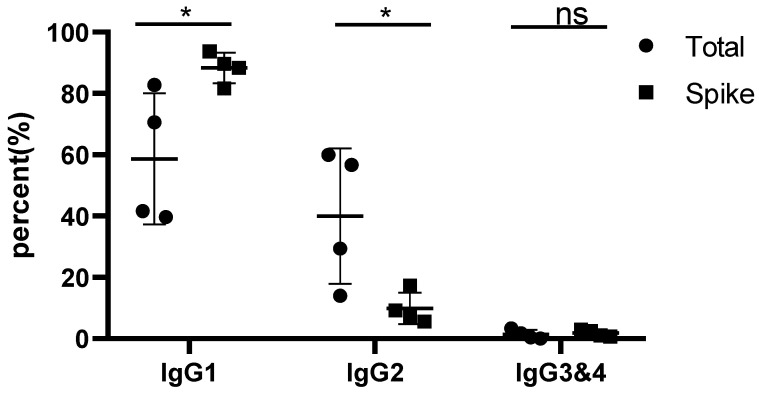
Percentage of IgG1 in Spike protein-specific immunoglobulins. Spike-specific immunoglobulins (Spike) were adsorbed by plate-bound recombinant trimeric Spike proteins and analyzed by LC-MS and quantified by XIC AUC. Protein A/G-adsorbed total serum immunoglobulins (Total) were also analyzed. Note that the IgG3 and IgG4 subclasses of rhesus monkey were summarized together due to identical characteristic peptide fragments after trypsin digestion. “*” means significant difference (*p* < 0.05); “ns” means no significant difference.

In all monkeys vaccinated by Spike trimers containing PIKA adjuvant, IgG1 was detected as the major antibody subclass (Figure 1 and Supplementary Table S1). The average percentage of Spike protein-adsorbed IgG1 increased to 88.3% of four IgG subclasses, while the percentage of IgG1 in protein A-adsorbed serum IgGs was only about 60% of the four IgG subclasses. As human Th1 cells and IFN-γ responses are associated withhigh levels of IgG1 and IgG3, low levels of IgG2, and undetectable IgG4 antibody levels, the dominance of the IgG1 subclass induced by use of PIKA adjuvant indicates a Th1-biased CD4 T cell response. 

Spike-specific immunoglobulins (Spike) were adsorbed by plate-bound recombinant trimeric Spike proteins, analyzed by LC-MS, and quantified by XIC AUC. Total serum immunoglobulins (Total) were also analyzed. 

As the unique adjuvant that induces the dominant IgG1 subclass of antibodies, the mechanism that PIKA adjuvant (polyI:C) selectively induced through IgG1 class switch remains to be studied. IgG1 is most cytotoxic IgG subclass, followed by IgG3. IL27, Ets1, and Stat1 were reported to be required for induction of mouse IgG2a, the counterpart of monkey and human IgG1 [27,28]. The Steinman Group reported polyI:C as a unique adjuvant stimulating CD4-Th1 response with a mechanism different from other adjuvants. The IFN-AR receptor was directly required for DCs to respond to poly IC. The adjuvant action of poly I:C requires a widespread innate type I IFN response dependent on STAT1 and mda5 signaling molecules, which activate dendritic cells in a TLR3-dependent mechanism [29]. Francica et al. studied non-human primates immunized with a glycoprotein 140 HIV envelope protein (Env) and compared different types of adjuvants, including insoluble aluminum salts (alum), MF59, adjuvant nanoemulsion (ANE) coformulated with or without Toll-like receptor 4 (TLR4) and 7 agonists, polyinosinic-polycytidylic acid:poly-L-lysine, carboxymethylcellulose (pIC:LC), and immune-stimulating complexes. Antiviral/IFN gene signatures were found to correlate with Fc-receptor binding across all adjuvant groups [30]. 

The effect of a few adjuvants on IgG subclasses have been recently reported in a few clinical trials. Farkash et al. studied Spike RBD-adsorbed IgGs and reported IgG1 as the predominant subclass, followed by IgG3 and IgG2 plus negligible IgG4. Importantly, individuals in the >60 years old Group displayed decreased (IgG1 + IgG3): (IgG2 + IgG4) ratios as compared to the <60 years old individuals due to lower IgG1 and IgG3 levels; however, both groups displayed similar IgG2 responses [31]. Buhre et al. compared anti-Spike antibody responses in mRNA vaccines and adenovirus-based vaccines. mRNA vaccines induced anti-Spike IgG1 followed by IgG3 and IgG2 and hardly detectable IgG4, whereas the AZD1222 adenovirus vaccine induced IgG1 and IgG3; however, it hardly induced any IgG2 and IgG4 [32]. Compared to above studies, PIKA adjuvant used in our study induced predominant IgG1, followed by IgG2, IgG3 and IgG4. The pattern of IgG subclasses clearly indicate the roles of different cellular and molecular mechanisms by PIKA adjuvant in regulating IgG class switch. 

Since three-to-four-year-old healthy Cynomolgus monkeys were used in this study, it remains unknown whether the PIKA COVID-19 vaccine may induce Th1-dominant CD4 and antibody responses in patients with underlying health conditions. Bigenwald studied patients with hematological malignancies vaccinated by mRNA vaccines, and reported that only S1-RBD-specific Th1 responses were associated with protection against SARS-CoV-2 infection, while Th2 responses or anti-S1-RBD IgG titers failed to correlate with protection [33]. Impaired Th1 responses and vaccine efficacy were also reported in type-2 diabetes patients vaccinated by either mRNA or inactivated SARS-CoV-2 vaccines [34]. 

### 2.2. PIKA Adjuvant Induced Fully Core-Fucosylated IgG Subclasses

In all monkeys vaccinated by Spike trimers containing PIKA adjuvant, fucosylation of S-trimer-adsorbed IgG1 was above 99% (Supplementary Tables S2 and S3), while the percentage of fucosylation in total serum IgG1 varies between 91% and 99%. Defective fucosylation was found to be between 1% and 8% of total serum IgG1, while only 1%, on average, for S-trimer-adsorbed IgG1 (Supplementary Table S4). 

Afucosylated IgG1 was previously reported in 5–10% of healthy individuals and had a yet unknown function [19]. In COVID-19 patients, high levels of afucosylated IgG1 were associated with cytokine storm and severe diseases [18,19]. The afucosylated IgG1 showed a more than 40-fold increase in binding to Fc gamma receptor 3A (FCGR3A), which overstimulated monocytes and other effector cells. Through antibody glycoengineering, we have prepared IgG1 monoclonal antibodies (S309) with homogeneous G2F glycosylation that inhibit the cytokine release of monocytes by afucosylated IgG1-Spike immune complexes (Dapeng Zhou, Feng Tang and Wei Huang, data to be published elsewhere). 

The effect of adjuvants on Fc glycosylation of every IgG subclass have been reported recently as the technologies for analyzing the glycosylation profile of IgG Fc glycopeptides emerge. Selman et al. studied the M59 adjuvant used for influenza vaccine and the alum adjuvant used for tetanus vaccine. No significant differences were found in fucosylation profiles of vaccine-adsorbed IgG subclasses, though increased galactosylation and sialylation were reported [35]. Van Coillie et al. reported that mRNA vaccine transiently induced afucosylated IgGs in naïve individuals after first and second dose of vaccines [20]. Farkash et al. found that mRNA vaccine-induced anti-SARS-CoV-2 IgG3 fucosylation decreased over time, while IgG1 fucosylation increased [31]. Buhre et al. compared anti-Spike antibody responses in mRNA vaccines and adenovirus-based vaccines. A temporarily afucosylation of IgG1 was reported after first dose of immunization by mRNA vaccine with the fucosylation levels as low as 80%; however, the fucosylation level steadily increased in the next few weeks to about 98%. One immunization with AZD1222 induced IgG1 subclass with >95% fucosylation initially; this prevalence later increased to 98%. The galactosylation and sialylation of IgG1 was found to decrease over time for both mRNA and adenovirus-based vaccines [32]. Our studies in rhesus monkeys clearly showed that PIKA adjuvant induced >98% core-fucosylated IgG1, while the dynamic changes of IgG1 fucosylation and other glycosylation profiles are being further studied. 

Independent groups have reported that the mechanism for incomplete fucosylation of the IgG1 is caused by decreased transcription of alpha1,6 fucosyltransferese 8 (FUT8, 18–19). Teyleart et al. reported KLF15 as a putative repressor of FUT8 transcription in rat hybridoma cells [36]. GWAS study of human N-glycome identified HIF1alpha as a regulator of FUT8 in hepatic cells, while its function in B cells and plasma cells is unclear [37]. Yamaguchi et al. identified binding sites for some transcription factors, such as bHLH, cMyb and GATA-1, as well as a TATA-box in the human FUT8 promoter region [38]. Klaric et al. reported that knockdown of IKZF1 decreases the expression of fucosyltransferase FUT8 [39]. The exact signaling pathway leading to decreased FUT8 transcription in plasma cells is unknown. Genetic pre-disposition might also exist in the signaling pathway. Singh et al. reported reduced induction of SARS-CoV-2 specific T cell responses in children with multisystem inflammatory syndrome compared with COVID-19. In contrast, antibody responses were similar among convalescent COVID-19 and MIS-C. Cytokine production by CD4 Th1 cells was reduced in MISC patients; whether such a defect caused reduced a percentage of the IgG1 subclass or glycosylation is unknown [40]. 

Our study clearly indicates that PIKA adjuvant can rapidly induce fully fucosylated IgG1. Although PIKA adjuvant induced potent CD4 Th1 responses, whether the PIKA COVID-19 vaccine may induce full fucosylation in infected individuals with high afucosylated IgG1 is unknown; that topic is focus of our ongoing studies. 

## 3. Methods

### 3.1. Immunization of Cynomolgus Monkey

Banked sera of vaccinated Cynomolgus monkeys were provided by Yisheng Biopharma Ltd., Beijing, China. Cynomolgus monkeys aged between 2.9 and 4.6 years were immunized by the PIKA COVID-19 vaccine as described [8]. The vaccine was administered via intramuscular route at day 1, 8, 15 and 29. Every dose contained 30 μg of recombinant trimeric Spike proteins and 3 mg PIKA (polyI:C) adjuvant. Blood was drawn at day 57 for measuring immunoglobulins. 

### 3.2. Purification of Immunoglobulins Specific to Trimeric Spike Proteins

Recombinant Spike protein trimers of SARS-CoV-2 (Wuhan-01 strain) were prepared as described [7]. Recombinant S-trimer protein solution was coated on ELISA plate (Thermo Scientific, Waltham, MA, F96 MAXISORP NUNC-IMMUNO PLATE) at 2 μg/mL in PBS (100 μL per well). The coated plates were incubated for 2 h at 37 °C. The supernatant was discarded and the plates were washed with 200 μL PBS containing 0.05% Tween three times. Coated plates were blocked with PBS containing 1% BSA (200 μL per well) and incubated for 1 h at 37 °C. The plates were washed with PBS containing 0.05% Tween three times. Monkey sera, five times diluted in PBS, were added (100 μL per well) and incubated for 12 h at 4 °C. Supernatant was discarded and plates were washed for five times with 200 μL PBS containing 0.05% Tween. To elute the adsorbed immunoglobulins, 0.1 M Glycine- HC l (pH 2.6) was added (100 μL per well) for 15 min with shaking. The eluted immunoglobulins were neutralized by 1 M Tris (pH 8.0, 100 μL per well), pooled, and desalted with microcentrifugation unit (Thermo scientific, Pierce^TM^ Protein Concentrator PES 10K MWCO). Pooled immunoglobulin eluates were washed twice by adding 5 mL of H_2_O. The protein concentration was determined by a BCA protein quantification kit. About 1 microgram of Spike-specific immunoglobulins was typically harvested from 100 μL monkey serum. 

### 3.3. Protein Digestion

The enriched IgG samples were precipitated with cold acetone, and the precipitates were dissolved by 8 M Urea. The proteins were reduced and alkylated by adding 5 mM TCEP and 10 mM IAA, before being digested by trypsin (at the weight ratio of 1:50) overnight at 37 °C. Peptides were desalted with MonoSpinTM C18 column (GL Science, Tokyo, Japan) and dried by SpeedVac, reconstituted with 0.1% formic acid.

### 3.4. LC/MS/MS Analysis

The desalted peptides were loaded onto a homemade 20 cm-long pulled-tip analytical column (ReproSil-Pur C18-AQ 1.9 μm resin, Dr. Maisch GmbH, 360 μm OD × 75 μm ID) connected to a nano Elute HPLC and Bruker timsTOF Pro2 for mass spectrometry analysis. The mobile phases and elution gradients used for separation were set as follows: 0–24 min, 5–15% solvent B (0.1% formic acid in ACN); 24–25 min, 15–35% B; 25–26 min, 35–80% B; 26–30 min, 80% B (buffer A: 0.1% FA in water and buffer B: 0.1% FA in 80% Acetonitrile), at a flow rate of 300 nL/min. Peptides eluted from the LC column were directly electro-sprayed into the mass spectrometer with the application of a distal 1.5 kV spray voltage. Survey full-scan MS spectra and MS2 spectra (from *m*/*z* 100–4000) were acquired in the TOF analyzer with PASEF mode. The cycle time was set as 3 s. The number of Parallel Accumulation–Serial Fragmentation (PASEF) ramps was set at 6. Target intensity and intensity threshold were set at 100 K and 1000, respectively. 

### 3.5. Data Processing and Analysis

All the acquired MS/MS and MS data were interpreted and searched by the GlycanFinder algorithm for glycopeptides identification and quantification based on XIC AUC. The parameters for database search of intact glycopeptide were as follows: mass tolerance for precursors and fragment ions were set as ±20 and ±40 ppm, respectively. The enzyme was chosen as fully trypsin digested. Maximal missed cleavage was 1. Fixed modification was carbamidomethylation on all Cys residues (C +57.022 Da). Variable modifications contained oxidation on Met (M +15.995 Da) and deamidation on NQ (+0.98 Da). Moreover, the identified N-glycopeptides were further examined manually to verify the accuracy. The glycopeptides were quantified by GlycanFinder based on XIC AUC.

## Data Availability

Data is contained within the article or supplementary material.

## Supplementary Materials

The following supporting information can be downloaded at: https://www.mdpi.com/article/10.3390/vaccines11040827/s1, Table S1: IgG subclasses as measured by LC-MS and quantified by XIC AUC. IgG subclasses were analyzed according to trypsin-digested peptide fragments. Table S2: All IgG glycopeptides as measured by LC–MS and quantified by XIC AUC. Fc peptide ETQYNSTYR modified by all types of glycans including both fucosylated and afucosylated were analyzed. Table S3: Percentage of fucosylated IgG subclasses as measured by LC–MS and quantified by XIC AUC. Table S4: Percentage of afucosylated IgG subclasses as measured by LC–MS and quantified by XIC AUC.

## Abbreviations

FUT8: alpha1,6fucosyltransferase 8; LC-MS, Liquid chromatography-mass spectrometry; XIC, extracted ion chromatogram; AUC, area-under-the-curve.

## References

[1] Shinde V., Bhikha S., Hoosain Z., Archary M., Bhorat Q., Fairlie L., Lalloo U., Masilela M.S.L., Moodley D., Hanley S. (2021). Efficacy of NVX-CoV2373 COVID-19 Vaccine against the B.1.351 Variant. N. Engl. J. Med..

[2] Dunkle L.M., Kotloff K.L., Gay C.L., Áñez G., Adelglass J.M., Barrat Hernández A.Q., Harper W.L., Duncanson D.M., McArthur M.A., Florescu D.F. (2022). Efficacy and Safety of NVX-CoV2373 in Adults in the United States and Mexico. N. Engl. J. Med..

[3] Bravo L., Smolenov I., Han H.H., Li P., Hosain R., Rockhold F., Clemens S.A.C., Roa C., Borja-Tabora C., Quinsaat A. (2022). Efficacy of the adjuvanted subunit protein COVID-19 vaccine, SCB-2019: A phase 2 and 3 multicentre, double-blind, randomised, placebo-controlled trial. Lancet.

[4] Tadesse B.T., Bravo L., Marks F., Aziz A.B., You Y.A., Sugimoto J., Li P., Garcia J., Rockhold F., Clemens R. (2022). Impact of vaccination with SCB-2019 COVID-19 vaccine on transmission of SARS-CoV-2 infection: A household contact study in the Philippines. Clin. Infect. Dis..

[5] Hannawi S., Saifeldin L., Abuquta A., Alamadi A., Mahmoud S.A., Hassan A., Liu D., Yan L., Xie L. (2023). Safety and immunogenicity of a bivalent SARS-CoV-2 protein booster vaccine, SCTV01C, in adults previously vaccinated with mRNA vaccine: A randomized, double-blind, placebo-controlled phase 1/2 clinical trial. eBioMedicine.

[6] Hannawi S., Saifeldin L., Abuquta A., Alamadi A., Mahmoud S.A., Li J., Chen Y., Xie L. (2023). Safety and immunogenicity of a bivalent SARS-CoV-2 protein booster vaccine, SCTV01C in adults previously vaccinated with inactivated vaccine: A randomized, double-blind, placebo-controlled phase 1/2 clinical trial. J. Infect..

[7] Tong J., Zhu C., Lai H., Feng C., Zhou D. (2021). Potent Neutralization Antibodies Induced by a Recombinant Trimeric Spike Protein Vaccine Candidate Containing PIKA Adjuvant for COVID-19. Vaccines.

[8] Liu Y., Dai L., Feng X., Gao R., Zhang N., Wang B., Han J., Zou Q., Guo X., Zhu H. (2021). Fast and long-lasting immune response to S-trimer COVID-19 vaccineadjuvanted by PIKA. Mol. Biomed..

[9] Liu Y., Tan L.H., Zhang N., Zhang Y., Mojares Z.R. (2022). Safety, Tolerability, and Immunogenicity of PIKA-Adjuvanted Recombinant SARS-CoV-2 Spike (S) Protein Subunit Vaccine in Healthy Adults: Interim results of an open-label and randomised Phase 1 clinical trial. medRxiv.

[10] Torales J., Cuenca-Torres O., Barrios L., Armoa-Garcia L., Estigarribia G., Sanabria G., Lin M.Y., Antonio Estrada J., Estephan L., Cheng H.Y. (2023). An evaluation of the safety and immunogenicity of MVC-COV1901: Results of an interim analysis of a phase III, parallel group, randomized, double-blind, active-controlled immunobridging study in Paraguay. Vaccine.

[11] Keech C., Albert G., Cho I., Robertson A., Reed P., Neal S., Plested J.S., Zhu M., Cloney-Clark S., Zhou H. (2020). Phase 1–2 Trial of a SARS-CoV-2 Recombinant Spike Protein Nanoparticle Vaccine. N. Engl. J. Med..

[12] Rydyznski Moderbacher C., Kim C., Mateus J., Plested J., Zhu M., Cloney-Clark S., Weiskopf D., Sette A., Fries L., Glenn G. (2022). NVX-CoV2373 vaccination induces functional SARS-CoV-2-specific CD4+ and CD8+ T cell responses. J. Clin. Investig..

[13] Painter M.M., Mathew D., Goel R.R., Apostolidis S.A., Pattekar A., Kuthuru O., Baxter A.E., Herati R.S., Oldridge D.A., Gouma S. (2021). Rapid induction of antigen-specific CD4+ T cells is associated with coordinated humoral and cellular immunity to SARS-CoV-2 mRNA vaccination. Immunity.

[14] Zhang B., Huo J., Huang Y., Teo S.Y., Duan K., Li Y., Toh L.K., Lam K.P., Xu S. (2022). mRNA Booster Vaccination Enhances Antibody Responses against SARS-CoV2 Omicron Variant in Individuals Primed with mRNA or Inactivated Virus Vaccines. Vaccines.

[15] van Doremalen N., Lambe T., Spencer A., Belij-Rammerstorfer S., Purushotham J.N., Port J.R., Avanzato V.A., Bushmaker T., Flaxman A., Ulaszewska M. (2020). ChAdOx1 nCoV-19 vaccine prevents SARS-CoV-2 pneumonia in *Rhesus macaques*. Nature.

[16] Ewer K.J., Barrett J.R., Belij-Rammerstorfer S., Sharpe H., Makinson R., Morter R., Flaxman A., Wright D., Bellamy D., Bittaye M. (2021). T cell and antibody responses induced by a single dose of ChAdOx1 nCoV-19 (AZD1222) vaccine in a phase 1/2 clinical trial. Nat. Med..

[17] Ferrara C., Grau S., Jäger C., Sondermann P., Brünker P., Waldhauer I., Hennig M., Ruf A., Rufer A.C., Stihle M. (2011). Unique carbohydrate-carbohydrate interactions are required for high affinity binding between FcγRIII and antibodies lacking core fucose. Proc. Natl. Acad. Sci. USA.

[18] Chakraborty S., Gonzalez J., Edwards K., Mallajosyula V., Buzzanco A.S., Sherwood R., Buffone C., Kathale N., Providenza S., Xie M.M. (2019). Proinflammatory IgG Fc structures in patients with severe COVID-19. Nat. Immunol..

[19] Larsen M.D., de Graaf E.L., Sonneveld M.E., Plomp H.R., Nouta J., Hoepel W., Chen H.-J., Linty F., Visser R., Brinkhaus M. (2021). Afucosylated IgG characterizes enveloped viral responses and correlates with COVID-19 severity. Science.

[20] Van Coillie J., Pongracz T., Rahmöller J., Chen H.J., Geyer C.E., van Vught L.A., Buhre J.S., Šuštić T., van Osch T.L.J., Steenhuis M. (2023). The BNT162b2 mRNA SARS-CoV-2 vaccine induces transient afucosylated IgG1 in naive but not in antigen-experienced vaccinees. eBioMedicine.

[21] Tanabe M., Kurita-Taniguchi M., Takeuchi K., Takeda M., Ayata M., Ogura H., Matsumoto M., Seya T. (2003). Mechanism of up-regulation of human Toll-like receptor 3 secondary to infection of measles virus-attenuated strains. Biochem. Biophys. Res. Commun..

[22] Goriely S., Molle C., Nguyen M., Albarani V., Haddou N.O., Lin R., De Wit D., Flamand V., Willems F., Goldman M. (2006). Interferon regulatory factor 3 is involved in Toll-like receptor 4 (TLR4)- and TLR3-induced IL-12p35 gene activation. Blood.

[23] Gitlin L., Barchet W., Gilfillan S., Cella M., Beutler B., Flavell R.A., Diamond M.S., Colonna M. (2006). Essential role of mda-5 in type I IFN responses to polyriboinosinic:polyribocytidylic acid and encephalomyocarditis picornavirus. Proc. Natl. Acad. Sci. USA.

[24] Kalimuddin S., Wijaya L., Chan Y.F.Z., Wong A.W.L., Oh H.M.L., Wang L.F., Kassim J.A., Zhao J., Shi Z., Low J.G. (2017). A phase II randomized study to determine the safety and immunogenicity of the novel PIKA rabies vaccine containing the PIKA adjuvant using an accelerated regimen. Vaccine.

[25] Wijaya L., Tham C.Y.L., Chan Y.F.Z., Wong A.W.L., Li L.T., Wang L.F., Bertoletti A., Low J.G. (2017). An accelerated rabies vaccine schedule based on toll-like receptor 3 (TLR3) agonist PIKA adjuvant augments rabies virus specific antibody and T cell response in healthy adult volunteers. Vaccine.

[26] Scinicariello F., Engleman C.N., Jayashankar L., McClure H.M., Attanasio R. (2004). Rhesus macaque antibody molecules: Sequences and heterogeneity of alpha and gamma constant regions. Immunology.

[27] Yoshimoto T., Okada K., Morishima N., Kamiya S., Owaki T., Asakawa M., Iwakura Y., Fukai F., Mizuguchi J. (2004). Induction of IgG2a class switching in B cells by IL-27. J. Immunol..

[28] Nguyen H.V., Mouly E., Chemin K., Luinaud R., Despres R., Fermand J.P., Arnulf B., Bories J.C. (2012). The Ets-1 transcription factor is required for Stat1-mediated T-bet expression and IgG2a class switching in mouse B cells. Blood.

[29] Longhi M.P., Trumpfheller C., Idoyaga J., Caskey M., Matos I., Kluger C., Salazar A.M., Colonna M., Steinman R.M. (2009). Dendritic cells require a systemic type I interferon response to mature and induce CD4+ Th1 immunity with poly IC as adjuvant. J. Exp. Med..

[30] Francica J.R., Zak D.E., Linde C., Siena E., Johnson C., Juraska M., Yates N.L., Gunn B., De Gregorio E., Flynn B.J. (2017). Innate transcriptional effects by adjuvants on the magnitude, quality, and durability of HIV envelope responses in NHPs. Blood Adv..

[31] Farkash I., Feferman T., Cohen-Saban N., Avraham Y., Morgenstern D., Mayuni G., Barth N., Lustig Y., Miller L., Shouval D.S. (2021). Anti-SARS-CoV-2 antibodies elicited by COVID-19 mRNA vaccine exhibit a unique glycosylation pattern. Cell Rep..

[32] Buhre J.S., Pongracz T., Künsting I., Lixenfeld A.S., Wang W., Nouta J., Lehrian S., Schmelter F., Lunding H.B., Dühring L. (2023). mRNA vaccines against SARS-CoV-2 induce comparably low long-term IgG Fc galactosylation and sialylation levels but increasing long-term IgG4 responses compared to an adenovirus-based vaccine. Front. Immunol..

[33] Bigenwald C., Haddad Y., Thelemaque C., Carrier A., Birebent R., Ly P., Flament C., Lahmar I., de Sousa E., Maeurer M. (2023). RBD- specific Th1 responses are associated with vaccine-induced protection against SARS-CoV-2 infection in patients with hematological malignancies. Oncoimmunology.

[34] Lee C.H., Gray V., Teo J.M.N., Tam A.R., Fong C.H., Lui D.T., Pang P., Chan K.H., Hung I.F., Tan K.C. (2022). Comparing the B and T cell-mediated immune responses in patients with type 2 diabetes receiving mRNA or inactivated COVID-19 vaccines. Front. Immunol..

[35] Selman M.H., de Jong S.E., Soonawala D., Kroon F.P., Adegnika A.A., Deelder A.M., Hokke C.H., Yazdanbakhsh M., Wuhrer M. (2012). Changes in antigen-specific IgG1 Fc N-glycosylation upon influenza and tetanus vaccination. Mol. Cell Proteom..

[36] Teylaert B., Meurice E., Bobowski M., Harduin-Lepers A., Gaucher C., Fontayne A., Jorieux S., Delannoy P. (2011). Molecular cloning, characterization, genomic organization and promoter analysis of the α1,6-fucosyltransferase gene (fut8) expressed in the rat hybridoma cell line YB2/0. BMC Biotechnol..

[37] Lauc G., Essafi A., Huffman J.E., Hayward C., Knežević A., Kattla J.J., Polašek O., Gornik O., Vitart V., Abrahams J.L. (2010). Genomics meets glycomics-the first GWAS study of human N-Glycome identifies HNF1α as a master regulator of plasma protein fucosylation. PLoS Genet..

[38] Yamaguchi Y., Ikeda Y., Takahashi T., Ihara H., Tanaka T., Sasho C., Uozumi N., Yanagidani S., Inoue S., Fujii J. (2000). Genomic structure and promoter analysis of the human alpha1, 6-fucosyltransferase gene (FUT8). Glycobiology.

[39] Klarić L., Tsepilov Y.A., Stanton C.M., Mangino M., Sikka T.T., Esko T., Pakhomov E., Salo P., Deelen J., McGurnaghan S.J. (2020). Glycosylation of immunoglobulin G is regulated by a large network of genes pleiotropic with inflammatory diseases. Sci. Adv..

[40] Singh V., Obregon-Perko V., Lapp S.A., Horner A.M., Brooks A., Macoy L., Hussaini L., Lu A., Gibson T., Silvestri G. (2022). Limited induction of SARS-CoV-2-specific T cell responses in children with multisystem inflammatory syndrome compared with COVID-19. JCI Insight..

